# Gastric endoscopic submucosal dissection through a gastrostomy using a newly developed thin endoscope

**DOI:** 10.1055/a-2333-9313

**Published:** 2024-06-12

**Authors:** Satoki Shichijo, Mori Hitoshi, Koji Higashino, Noriya Uedo, Tomoki Michida

**Affiliations:** 1 53312 Gastrointestinal Oncology, Osaka International Cancer Institute, Osaka, Japan

A 76-year-old man underwent follow-up endoscopy after undergoing curative endoscopic submucosal dissection (ESD) for esophageal cancer 1 . He had a past history of advanced pharyngeal cancer, which had been treated with chemoradiotherapy, and had a percutaneous endoscopic gastrostomy because of persistent trismus ( Fig. 1 ). The follow-up endoscopy, performed via transnasal endoscopy, revealed a 6-mm depressed lesion in the lesser curvature of the antrum, and a biopsy confirmed adenocarcinoma ( Fig. 2 ). ESD using a newly developed endoscope 2 3 was performed to treat the gastric cancer.

**Fig. 1:**
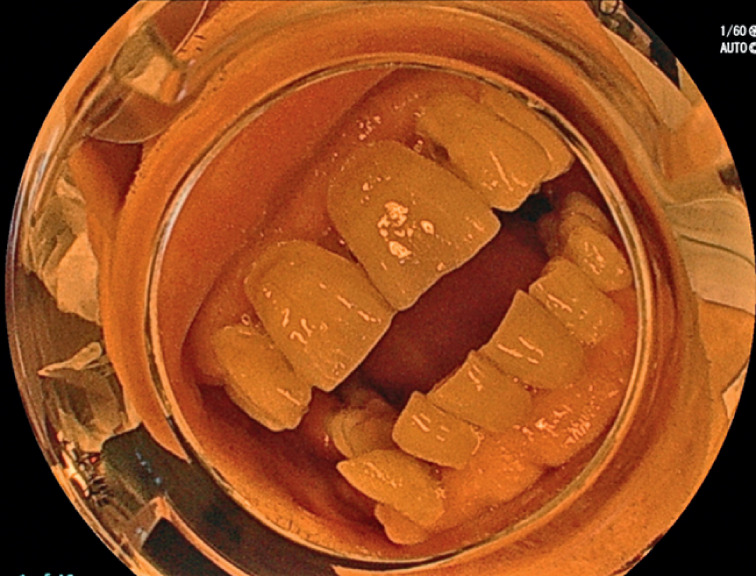
Photograph showing persisting trismus after chemoradiotherapy for advanced pharyngeal cancer.

**Fig. 2:**
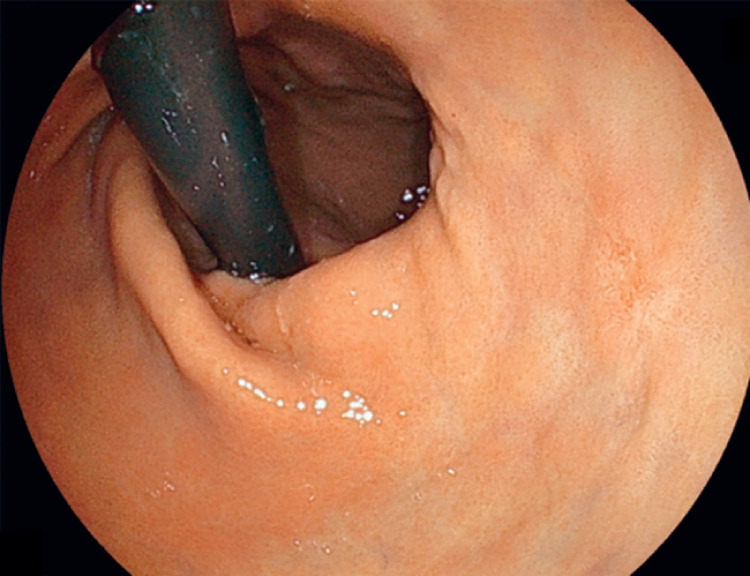
Endoscopic images showing a slightly depressed lesion at the lesser curvature of the antrum viewed on transnasal endoscopy.

First, the catheter through the gastrostomy was removed and an endoscope with a diameter of 7.9 mm (EG-840TP; Fujifilm, Tokyo, Japan) was inserted through the gastrostomy ( Fig. 3 ; Video 1 ). Circumferential marking, mucosal incision, and circumferential incision were performed, and submucosal dissection was subsequently performed until the tumor was resected en bloc ( Fig. 4 ), taking 9 minutes. The lesion was retrieved through the gastrostomy, and a new catheter was placed into the gastrostomy using a guidewire. The final pathologic diagnosis was a 6×6-mm, 0–IIc, well-differentiated tubular adenocarcinoma, pT1a, pUL0, ly0, v0, pHM0, pVM0.

Gastric endoscopic submucosal dissection is performed through a gastrostomy using a newly developed thin endoscope.Video 1

**Fig. 3:**
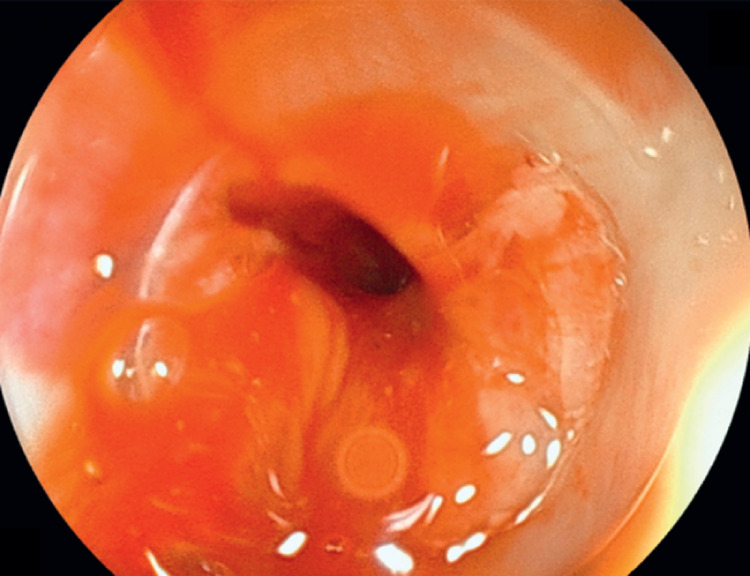
An endoscope was inserted through gastrostomy.

**Fig. 4:**
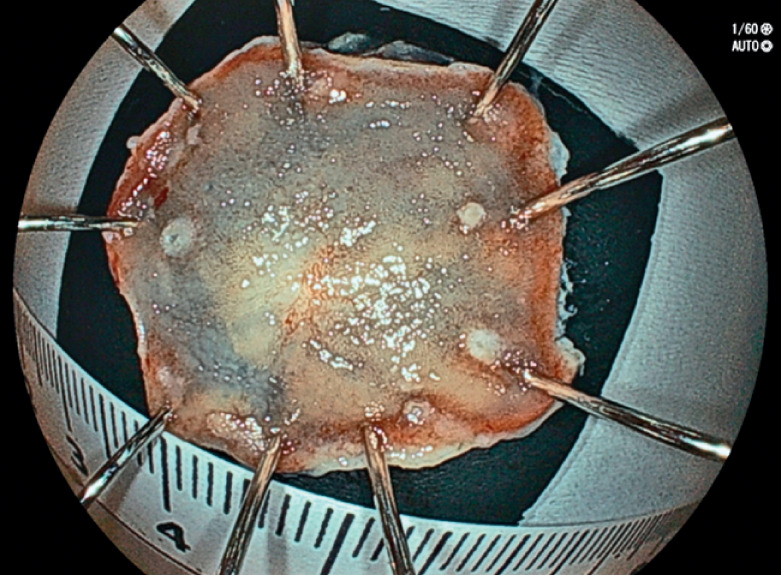
Macroscopic appearance of the lesion, which was resected en bloc.

Although the newly developed endoscope has a large working channel of 3.2 mm and offers wide angles (up 210°; down 160°), its small width of 7.9 mm enabled efficient ESD to be performed through the gastrostomy without dilation 1 .

Endoscopy_UCTN_Code_TTT_1AO_2AG_3AD

## Notes

Endoscopy E-Videos https://eref.thieme.de/e-videos
*E-Videos* is an open access online section of the journal *Endoscopy* , reporting on interesting cases and new techniques in gastroenterological endoscopy. All papers include a high-quality video and are published with a Creative Commons CC-BY license. Endoscopy E-Videos qualify for HINARI discounts and waivers and eligibility is automatically checked during the submission process. We grant 100% waivers to articles whose corresponding authors are based in Group A countries and 50% waivers to those who are based in Group B countries as classified by Research4Life (see: https://www.research4life.org/access/eligibility/ ). This section has its own submission website at https://mc.manuscriptcentral.com/e-videos .
